# Society Meetings

**Published:** 1914-03

**Authors:** 


### SOCIETY MEETINGS.

*Brief reports and announcements of meetings of societies of Western New York and those of general scope are requested from Secretaries. Copy should be on hand the fifteenth of each month. Full transactions will be published at cost of composition.*

---

THE ROCHESTER ACADEMY OF MEDICINE, Section 2, Surgery and Surgical Specialties, met at the Academy rooms, February 11. Dr. Lee Masten Francis of Buffalo spoke on "Sclero-Corneal Trephining," according to the method of Col. Elliott in the Operative Treatment of Glaucoma. A subscription dinner was tendered to Dr. Francis at the Rochester Club before the meeting.

---

THE ELMIRA ACADEMY OF MEDICINE met February 11. Dr. W. A. DeWitt of Blossburg, Pa., gave a paper on "Artificial Pneumothorax," and Dr. F. W. Ross "Reminiscences of Thirty Years of Practice in Elmira."

The Buffalo Academy of Medicine has held the following meetings: Pathology, January 27; Problems in Renal Pathology, Dr. George Baehr of Mt. Sinai Hospital, New York.

Surgery, February 3. Original Surgical Uses of the Bone and Cartilage Craft—a report of 230 cases, illustrated by lantern; Dr. Fred H. Albee, F. G. Hospital, New York. Discussion opened by Drs. Park, Bartow and Le Breton.

Medicine, February 10. The Heart in Some Infections, Dr. A. E. Woehnert; The Modern Conception of Gastric Neuroses, Dr. A. A. Jones.

On February 17 the program announced by the section of Obstetrics and Gynæcology was suspended by a special meeting of the entire academy, in memory of Dr. Roswell Park. Captain Harry R. Trick, President, opened the meeting with appropriate general remarks and addresses were made as follows: Dr. Thomas H. McKee, Dr. Park as a Teacher; Dr. Herman G. Matzinger, Dr. Park as an Author; Dr. Charles G. Stockton, the Personality of the Man; Dr. Edgar R. McGuire, Dr. Park's Influence on Surgery. Dr. Charles Cary, as chairman of a committee including Drs. Stockton, Edgar R. McGuire, Gaylord and Wende, presented resolutions which were adopted, ordered spread on the minutes and sent in duplicate to Dr. Park's family.

---

The Buffalo Medical and Surgical League had for its scientific program at the February meeting a paper entitled: "Gonorrhea," by Dr. Charles Bethune.

---

The Gross Medical Club of Buffalo held its annual meeting and banquet at the Hotel Statler, Friday afternoon and evening, February 19th. The election of officers resulted: Drs. A. G. Bennett, President; W. Warren Britt of Tonawanda, Vice-President; Chester C. Cott, Secretary. Dr. Bennett appointed the following committee on membership and to arrange the schedule for the coming year: Drs. Jas. E. King, J. Henry Dowd and Albert E. Perry. Dr. Geo. F. Cott gave a most enjoyable talk on his recent trip through Egypt, illustrating it with stereopticon views. The following cases were reported and freely discussed: Dr. Tyler, a boy eight or nine years old, ill with grip. Suddenly his temperature rose to 105, the next day it was normal but the next 104. Following this on the second day he was seized with a severe earache and four days later the right elbow joint became very painful, tender and swollen. This was aspirated a few days later and a large amount of pus found; the joint was irrigated with Tiersh solution. Was the joint condition due to the otitis? The ear is still discharging.

Dr. Jas. McLeod, a man aged 61, erysipelas of the scrotum, miscroscopic examination showed it to be streptococcus infection. To our great surprise the scrotum commenced sloughing, and soon the testes were entirely free of all covering. As soon as sloughing had ceased a new scrotum was formed by taking a flap from the right thigh. Except at one or two spots where there was a small amount of sloughing, healing took place by first intention and the patient has a fairly normal scrotum. These cases are very rare, the late Dr. Park having seen but three during his years of practice.

Dr. Dowd, a man aged 46, sent a sample of urine for examination with no history save that he was urinating frequently, no pain but loss of flesh and becoming very weak. Urinary examination showed Sp. G. 1008, large amount of albumin and pus, several hyaline caste, no sugar, bile, blood or spermatozoa, Phosphatic Index 90 per cent. minus, no evidence of tuberculosis but marked bacteriuria. Very little information being at hand to form an opinion Comp. Phos. Tonic was ordered in half teaspoonful doses three times a day half an hour after food. In about five weeks the man came to Buffalo and reported he had gained about twenty-two pounds, felt perfectly well but could not pass his urine for the past two weeks, excepting in the recumbent position. Examination of the urine at this time showed no albumin, only a few pus cells, no casts, an occasional uric acid crystal, P. I. now 15 per cent. minus. Further examination revealed prostate and vesicles normal, no stricture, organic in nature, but there was a markedly spasmodic contraction of the deep urethral muscles when the searcher approached that region. The sound quickly detected a stone and arrangements were made for an operation. Before leaving the office the man was requested to pass the water used for the examination, and to the surprise of both passed a stone (uric acid) as large as a kidney bean, over three-quarters of an inch long and three-quarters of an inch in circumference.

Dr. McKenney, a child aged eight months was suddenly seized with abdominal pain accompanied by marked shock. Careful examination revealed a possible intussusception, and, as the symptoms seemed to increase, operation was advised at once. The abdomen being opened it was found that the ilio-caecal valve, appendix and beginning of the ascending colon had telescoped into the ascending and transverse colon as far as the splenic flexure. A redection was done, removing at least six inches of intestine.

---

The Medical Society of the County of Erie held its regular meeting in the University of Buffalo on Monday, February 16th, 1914, with President John V. Woodruff in the chair.

The President announced the death of Dr. Roswell Park, which had occurred suddenly on February 15th, 1914, and resolutions of respect, moved by Dr. A. T. Lytle, were duly adopted by a unanimous rising vote.

The Secretary then read the minutes of the previous meeting and also of the council meetings, all of which were duly approved.

Dr. Grover W. Wende, Chairman of the Committee on Membership, then presented the applications of seventy-four candidates, each of whom was separately voted upon and admitted to membership.

At the conclusion of the election of so many new members, Dr. A. G. Bennett rose and stated that this was the first time in the history of the society that such a large number of new members had been elected at one time; that Dr. Wende had established a record, and that this is all the more important when we consider that, during the past five years, he had been instrumental in getting over 250 new members into the society, and especially when it is considered that practically every eligible practitioner within the county has, by this time, been elected to membership. He laid special stress also upon the revenue derived from such an additional membership, and concluded by saying that Dr. Wende was the logical candidate of this society for the office of President of the Medical Society of the State of New York. He, therefore, moved that it be the sense of this society that Dr. Grover W. Wende be its candidate for President of the Medical Society of the State of New York at the next annual meeting.

Dr. T. H. McKee seconded this motion with eulogistic remarks.

Motion was unanimously carried.

Dr. Julius Richter moved the following resolution:

"Resolved, That the Delegates from this Society be instructed to vote as a unit for the election of Dr. Grover W. Wende for President of the Medical Society of the State of New York."

Resolution was unanimously adopted.

Dr. Bennett moved that the Medical Society of the State of New York be invited to hold its 1915 meeting in Buffalo. Carried.

Dr. Lytle moved that a letter be sent to the delegates from the various County Societies to the State Society endorsing Dr. Wende's candidacy and including an invitation to hold the next meeting in Buffalo. Carried.

Dr. Bonnar moved that a vote of thanks be given to Dr. Wende for his excellent work as Chairman of the Committee on Membership. Carried.

Dr. A. L. Benedict, from the Committee on Necrology, reported to the Society the following deaths up to February 1, the obituaries being printed in the BUFFALO MEDICAL JOURNAL:

Hiram Dana Walker, University of Buffalo, 1864, died at Buffalo, February 21, 1913, aged 73 years.

Norman K. MacLeod, University of Toronto, 1903, died at Buffalo, April 3, 1913, aged 34 years.

Bernard F. Dennis, University of Buffalo, 1899, died at Oil City, Pa., July 31, 1913, aged 36 years.

Frederick C. Busch, University of Buffalo, 1897, died at Buffalo, January 3, 1914, aged 39 years.

This concluded the business session, after which Captain George H. Norton, Deputy Engineering Commissioner, Department of Public Works, gave a very interesting paper on "The Water Supply and Sewerage of Buffalo and the Public Health." The paper was thoroughly discussed by many members present, and at its conclusion a collation was served.

FRANKLIN C. GRAM, M. D.,
Secretary.

